# Supplementary material for: Acute kidney injury-attributable mortality in critically ill patients with sepsis
Source: PeerJ. 2022 Mar 25;10:e13184. doi: 10.7717/peerj.13184 (PMC8958971; doi:10.7717/peerj.13184)
Supplement: Supplemental Information 1 [file peerj-10-13184-s001.docx]

**Online Data Supplement**

**Title:** Acute kidney injury-attributable mortality in critically ill patients with sepsis

Zhiyi Wang^1,2^*, Jie Weng^1^*, Jinwen Yang^3^, Xiaoming Zhou^1^, Zhe Xu^4^, Ruonan Hou^1^, Zhiliang Zhou^1^, Liang Wang^5^, Chan Chen^3^, Shengwei Jin^6#^

^1^Department of General Practice, The Second Affiliated Hospital and Yuying Children's Hospital of Wenzhou Medical University, Wenzhou 325027, China

^2^Center for Health Assessment, Wenzhou Medical University

^3^Department of Geriatric Medicine, The First Affiliated Hospital, Wenzhou Medical University, Wenzhou 325000, China

^4^Department of Emergency Intensive Care Unit, The Second Affiliated Hospital and Yuying Children's Hospital of Wenzhou Medical University, Wenzhou 325027, China

^5^Department of Public Health, Robbins College of health and Human Sciences, Baylor University, Waco, TX, USA

^6^Department of Anesthesia and Critical Care, the Second Affiliated Hospital of Wenzhou Medical University, Wenzhou 325027, Zhejiang Province, China

**Equal contributors*

*#Correspondence to:* [*jinshengwei69@163.com*](mailto:jinshengwei69@163.com)

**Methods**

*Participants*

We studied adult patients from two distinct retrospective critically ill cohorts: (1) Medical Information Mart for Intensive Care IV (MIMIC IV) cohort and (2) Wenzhou cohort study[1]. The MIMIC IV cohort is a relational database containing comprehensive information on more than 250,000 patients hospitalized from 2008 to 2019 at a tertiary academic medical center (Beth Israel Deaconess Medical Center) in Boston, MA, USA. The Wenzhou cohort includes adult patients admitted from the emergency department to ICU at Second Affiliated Hospital and Yuying Children's Hospital of Wenzhou Medical University in Wenzhou, Zhejiang, China. The MIMIC IV was a third party publicly available database with pre-existing institutional review board (IRB) approval. Hence, IRB approval was not requested. Wenzhou cohort was approved by the Second Affiliated Hospital of Wenzhou Medical University IRB.

Enrollment in MIMIC IV occurred from 2008 to 2019. The study excluded patients who have history of kidney disease, multiple hospitalizations and ICU length of stay (LOS) less than 24 hours. Patients in MIMIC IV qualified as “critically ill” if they remained in the ICU (including Medical, Surgical or Cardiovascular ICU) for at least 1 day.

Enrollment in Wenzhou occurred from June 2012 to June 2020. For this analysis, we included patients admitted to the Emergency ICU, General ICU or Cardiovascular ICU directly from the emergency department. Patients in Wenzhou qualified as “critically ill” when an emergency physician requested an ICU admission and the patient was subsequently accepted and admitted to. Patients were excluded if they have history of kidney disease, multiple hospitalizations and ICU LOS less than 24 hours, had cardiac arrest prior to enrollment.

Primary Outcome and Additional Variables

Adult patients were enrolled based on the definition of sepsis-3 i.e. a known or suspected infection plus acute increase Sequential Organ Failure Assessment (SOFA) > 2 points for organ dysfunction [2, 3] from the MIMIC IV and Wenzhou cohorts. The study however excluded patients who have history of kidney disease, multiple hospitalizations (only the first hospitalization was considered) and ICU los less than 24 hours. Patients were defined as having AKI if they met the Kidney Disease: Improving Global Outcomes (KDIGO) serum creatinine diagnostic criteria for AKI within 7 days of hospital admission[4].  Baseline creatinine is defined as the lowest creatinine in the past 7 days after hospital admission. Shock was defined as use of vasopressors within the first 48 h of hospital admission. Respiratory failure was defined as the need for invasive mechanical ventilation.

KDIGO criteria for AKI

Serum creatinine criteria:

Stage 1: 1.5-1.9 times baseline OR ≥0.3 mg/dl (26.5 μmol/l) increase; Stage 2: 2.0-2.9 times baseline; Stage 3: 3 times baseline OR increase in serum creatinine to ≥ 4.0 mg/dl (≥353.6 μmol/l) OR Initiation of renal replacement therapy;

Urine output criteria:

Stage 1: <0.5 ml/kg/h for 6-12 hours; Stage 2: <0.5 ml/kg/h for ≥ 12 hours; Stage 3: < 0.3 ml/kg/h for ≥ 24 hours OR Anuria for ≥ 12 hours

Statistical Methods

**Attributable Mortality Fraction**

The attributable mortality fraction from AKI (AF_AKI_) is the proportion of deaths attributable to AKI among all deaths in patients with sepsis who developed AKI. To estimate the attributable mortality fraction from AKI in sepsis, we first take the number of people who had AKI and died and divide it by the total number who had AKI. We then compute the same fraction for those without AKI (with sepsis only). The attributable fraction is the difference between those two numbers divided by the first number (the fraction of those with AKI who died).  For example, if 27% of those with AKI died and 7% of those without AKI died, the attributable mortality fraction is the difference (20%) divided by 27%, or 74%.  Because we cannot directly measure the deaths attributable to AKI, the AF_AKI_ is adjusted based on indirect standardization, which computes the weighted average of stratum-specific estimates of mortality in the reference population, using weights from the study population[5, 6].  In other words, the AF_AKI_ is computed for each of the strata (in this case, defined by modified APS III), and then these stratum-specific estimates are combined to generate an overall AF_AKI_.  In each of these strata, we adjusted for the modified APS III. To estimate the population attributable fraction from AKI, we take the proportion of those who died (regardless of AKI status) and subtract the proportion without AKI who died. That difference is divided by the proportion of all who died.

Table S1. Association of AKI with mortality in unadjusted and adjusted models, MIMIC IV and Wenzhou cohorts including patients who meet urine output diagnostic criteria of KDIGO for AKI

| *MIMIC IV Logistic Regression Models (n=15610)* | *OR (95% CI)* | | *p* |
| --- | --- | --- | --- |
| Unadjusted model of AKI for hospital mortality | | 3.35 (2.97, 3.78) | <0.001 |
| Adjusted for modified APS III* | 1.81 (1.59, 2.07) | | <0.001 |
| Adjusted for modified SOFA score* | 1.95 (1.71, 2.22) | | <0.001 |
| Adjusted for modified LOSD* | 1.76 (1.55, 2.01) | | <0.001 |
| Adjusted for OASIS* | 1.61 (1.41, 1.84) | | <0.001 |
| Unadjusted model of AKI for ICU mortality | 4.53 (3.88, 5.33) | | <0.001 |
| Adjusted for modified APS III* | 2.22 (1.87, 2.65) | | <0.001 |
| Adjusted for modified SOFA score* | 2.41 (2.04, 2.86) | | <0.001 |
| Adjusted for modified LOSD* | 2.11 (1.79, 2.51) | | <0.001 |
| Adjusted for OASIS* | 1.93 (1.63, 2.30) | | <0.001 |
| *Wenzhou Logistic Regression Models (n=1341)* | *OR (95% CI)* | | *p* |
| Unadjusted model of AKI for hospital mortality | 2.28 (1.52, 3.53) | | <0.001 |
| Adjusted for modified APS III† | 1.43 (0.92, 2.29) | | 0.119 |
| Adjusted for modified SOFA score† | 1.42 (0.92, 2.26) | | 0.125 |
| Adjusted for modified LOSD† | 1.29 (0.83, 2.06) | | 0.268 |
| Adjusted for OASIS† | 1.25 (0.80, 2.00) | | 0.329 |
| Unadjusted model of AKI for ICU mortality | 4.36 (2.46, 8.44) | | <0.001 |
| Adjusted for modified APS III† | 2.63 (1.42, 5.27) | | 0.003 |
| Adjusted for modified SOFA score† | 2.59 (1.42, 5.15) | | 0.003 |
| Adjusted for modified LOSD† | 2.26 (1.22, 4.53) | | 0.014 |
| Adjusted for OASIS† | 2.20 (1.20, 4.37) | | 0.016 |
| Modified scores exclude points related to renal function.  *In addition to severity of illness variable listed in the table, adjusted models include age, gender, race, and shock  † In addition to severity of illness variable listed in the table, adjusted models include age, gender and shock | | | |

Table S2. Association of AKI with hospital and ICU length of stay in unadjusted and adjusted models, MIMIC IV and Wenzhou cohorts among only survivors

| *MIMIC IVI Linear Regression Models* | ***Coeff. (95% CI)*** | ***p*** |
| --- | --- | --- |
| Unadjusted model of AKI for hospital LOS (days) (n=13492) | 8.05 (7.65, 8.45) | <0.001 |
| Adjusted for modified APS III* | 5.99 (5.59, 6.40) | <0.001 |
| Adjusted for modified SOFA score* | 6.33 (5.92, 6.73) | <0.001 |
| Adjusted for modified LOSD* | 5.98 (5.57, 6.38) | <0.001 |
| Adjusted for OASIS* | 6.43 (6.02, 6.83) | <0.001 |
| Unadjusted model of AKI for ICU LOS (days) (n=13492) | 4.63 (4.43, 4.82) | <0.001 |
| Adjusted for modified APS III* | 3.17 (2.99, 3.36) | <0.001 |
| Adjusted for modified SOFA score* | 3.50 (3.30, 3.69) | <0.001 |
| Adjusted for modified LOSD* | 2.98 (2.80, 3.17) | <0.001 |
| Adjusted for OASIS* | 3.38 (3.19, 3.57) | <0.001 |
| *Wenzhou Linear Regression Models* | *Coeff. (95% CI)* | *p* |
| Unadjusted model of AKI for hospital LOS (days) (n=1186) | 5.87 (4.77, 6.96) | 0.004 |
| Adjusted for modified APS III† | 4.20 (3.11, 5.29) | <0.001 |
| Adjusted for modified SOFA score† | 4.59 (3.49, 5.69) | <0.001 |
| Adjusted for modified LOSD† | 4.46 (3.35, 5.56) | <0.001 |
| Adjusted for OASIS† | 4.62 (3.51, 5.73) | <0.001 |
| Unadjusted model of AKI for ICU LOS (days) (n=1186) | 3.95 (3.34, 4.57) | <0.001 |
| Adjusted for modified APS III† | 2.57 (1.99, 3.15) | <0.001 |
| Adjusted for modified SOFA score† | 2.98 (2.37, 3.59) | <0.001 |
| Adjusted for modified LOSD† | 2.55 (1.97, 3.14) | <0.001 |
| Adjusted for OASIS† | 2.86 (2.26, 3.47) | <0.001 |
| Modified scores exclude points related to renal function.  ***** In addition to severity of illness variable listed in the table, adjusted models include age, gender, race, and shock  **†** In addition to severity of illness variable listed in the table, adjusted models include age, gender and shock | | |

Table S3. Association of AKI with hospital and ICU length of stay in unadjusted and adjusted models, MIMC IV and Wenzhou cohorts among all patients

| *MIMIC IVI Linear Regression Models* | ***Coeff. (95% CI)*** | ***p*** |
| --- | --- | --- |
| Unadjusted model of AKI for hospital LOS (days) (n=15610) | 6.62 (6.25, 7.00) | <0.001 |
| Adjusted for modified APS III* | 5.45 (4.92, 5.98) | <0.001 |
| Adjusted for modified SOFA score* | 5.33 (4.93, 5.74) | <0.001 |
| Adjusted for modified LOSD* | 5.10 (4.69, 5.50) | <0.001 |
| Adjusted for OASIS* | 5.75 (5.35, 6.16) | <0.001 |
| Unadjusted model of AKI for ICU LOS (days) (n=15610) | 4.40 (4.21, 4.58) | <0.001 |
| Adjusted for modified APS III* | 2.90 (2.71, 3.08) | <0.001 |
| Adjusted for modified SOFA score* | 3.25 (3.06, 3.44) | <0.001 |
| Adjusted for modified LOSD* | 2.68 (2.50, 2.87) | <0.001 |
| Adjusted for OASIS* | 3.24 (3.05, 3.43) | <0.001 |
| *Wenzhou Linear Regression Models* | *Coeff. (95% CI)* | *p* |
| Unadjusted model of AKI for hospital LOS (days) (n=1341) | 4.97 (3.92, 6.01) | 0.004 |
| Adjusted for modified APS III† | 3.83 (2.74, 4.93) | <0.001 |
| Adjusted for modified SOFA score† | 3.96 (2.87, 5.04) | <0.001 |
| Adjusted for modified LOSD† | 4.04 (2.94, 5.15) | <0.001 |
| Adjusted for OASIS† | 4.04 (2.96, 5.14) | <0.001 |
| Unadjusted model of AKI for ICU LOS (days) (n=1341) | 4.16 (3.55, 4.76) | <0.001 |
| Adjusted for modified APS III† | 2.83 (2.22, 3.44) | <0.001 |
| Adjusted for modified SOFA score† | 3.15 (2.54, 3.77) | <0.001 |
| Adjusted for modified LOSD† | 2.71 (2.11, 3.31) | <0.001 |
| Adjusted for OASIS† | 3.07 (2.46, 3.68) | <0.001 |
| Modified scores exclude points related to renal function.  ***** In addition to severity of illness variable listed in the table, adjusted models include age, gender, race, and shock  **†** In addition to severity of illness variable listed in the table, adjusted models include age, gender and shock | | |

Table S4. ICU mortality stratified by severity of AKI

| *MIMIC IV ICU mortality* | | |
| --- | --- | --- |
| *Unadjusted model for ICU mortality* | *OR (95% CI)* | *p* |
| Stage 1 AKI (n=3187) | 4.12 (3.62, 4.79) | <0.001 |
| Stage 2 AKI (n=1117) | 7.81 (6.59, 9.23) | <0.001 |
| Stage 3 AKI (n=921) | 13.04 (11.03, 15.42) | <0.001 |
| Adjusted for modified APS III* |  |  |
| Stage 1 AKI | 2.31 (1.98, 2.69) | <0.001 |
| Stage 2 AKI | 3.35 (2.77, 4.05) | <0.001 |
| Stage 3 AKI | 4.34 (3.56, 5.27) | <0.001 |
| Adjusted for modified SOFA score* |  |  |
| Stage 1 AKI | 2.55 (2.20, 2.96) | <0.001 |
| Stage 2 AKI | 4.16 (3.46, 4.99) | <0.001 |
| Stage 3 AKI | 5.47 (4.52, 6.62) | <0.001 |
| Adjusted for modified LODS* |  |  |
| Stage 1 AKI | 2.37 (2.04, 2.75) | <0.001 |
| Stage 2 AKI | 3.44 (2.85, 4.14) | <0.001 |
| Stage 3 AKI | 4.83 (3.99, 5.85) | <0.001 |
| Adjusted for OASIS* |  |  |
| Stage 1 AKI | 2.45 (2.11, 2.85) | <0.001 |
| Stage 2 AKI | 3.87 (3.21, 4.65) | <0.001 |
| Stage 3 AKI | 5.03 (4.15, 6.08) | <0.001 |
| *Wenzhou ICU mortality* |  |  |
| *Unadjusted model for ICU mortality* | *OR (95% CI)* | *p* |
| Stage 1 AKI (n=323) | 5.11 (3.12, 8.54) | <0.001 |
| Stage 2 AKI (n=104) | 7.99 (4.27, 14.80) | <0.001 |
| Stage 3 AKI (n=67) | 12.50 (6.41, 24.13) | <0.001 |
| Adjusted for modified APS III* |  |  |
| Stage 1 AKI | 2.87 (1.67, 4.99) | <0.001 |
| Stage 2 AKI | 4.42 (2.24, 8.63) | <0.001 |
| Stage 3 AKI | 5.04 (2.33, 10.77) | <0.001 |
| Adjusted for modified SOFA score* |  |  |
| Stage 1 AKI | 3.37 (2.00, 5.79) | <0.001 |
| Stage 2 AKI | 4.38 (2.22, 8.53) | <0.001 |
| Stage 3 AKI | 5.81 (2.70, 12.32) | <0.001 |
| Adjusted for modified LODS* |  |  |
| Stage 1 AKI | 2.83 (1.66, 4.91) | <0.001 |
| Stage 2 AKI | 3.85 (1.92, 7.61) | <0.001 |
| Stage 3 AKI | 5.92 (2.76, 12.54) | <0.001 |
| Adjusted for OASIS* |  |  |
| Stage 1 AKI | 3.29 (1.95, 5.63) | <0.001 |
| Stage 2 AKI | 4.86 (2.49, 9.39) | <0.001 |
| Stage 3 AKI | 6.97 (3.29, 14.62) | <0.001 |
| Modified scores exclude points related to renal function  *In addition to severity of illness variable listed in the table, adjusted models include age, gender, race, and shock  † In addition to severity of illness variable listed in the table, adjusted models include age, gender and shock | | |

**References**

1. Zhou S, Zeng Z, Wei H, Sha T, An S: **Early combination of albumin with crystalloids administration might be beneficial for the survival of septic patients: a retrospective analysis from MIMIC-IV database.** *Ann Intensive Care* 2021, **11:**42.

2. Shankar-Hari M, Phillips GS, Levy ML, Seymour CW, Liu VX, Deutschman CS, Angus DC, Rubenfeld GD, Singer M: **Developing a New Definition and Assessing New Clinical Criteria for Septic Shock: For the Third International Consensus Definitions for Sepsis and Septic Shock (Sepsis-3).** *Jama* 2016, **315:**775-787.

3. Singer M, Deutschman CS, Seymour CW, Shankar-Hari M, Annane D, Bauer M, Bellomo R, Bernard GR, Chiche JD, Coopersmith CM, et al: **The Third International Consensus Definitions for Sepsis and Septic Shock (Sepsis-3).** *Jama* 2016, **315:**801-810.

4. Kellum. JA, Lameire. N, Aspelin. P, Barsoum. RS, Burdmann. EA, Goldstein. SL, Herzog. CA, Joannidis. M, Kribben. A, Levey. AS, et al: **Kidney disease: Improving global outcomes (KDIGO) acute kidney injury work group. KDIGO clinical practice guideline for acute kidney injury.** *Kidney Int Suppl* 2012, **2:**1-138.

5. van Vught LA, Klein Klouwenberg PM, Spitoni C, Scicluna BP, Wiewel MA, Horn J, Schultz MJ, Nürnberg P, Bonten MJ, Cremer OL, van der Poll T: **Incidence, Risk Factors, and Attributable Mortality of Secondary Infections in the Intensive Care Unit After Admission for Sepsis.** *Jama* 2016, **315:**1469-1479.

6. Auriemma CL, Zhuo H, Delucchi K, Deiss T, Liu T, Jauregui A, Ke S, Vessel K, Lippi M, Seeley E, et al: **Acute respiratory distress syndrome-attributable mortality in critically ill patients with sepsis.** *Intensive Care Med* 2020, **46:**1222-1231.
